# Renal Oncocytoma and Retroperitoneal Ancient Schwannoma: A Benign Mimic of Metastatic Renal Cell Carcinoma

**DOI:** 10.1155/2019/2561289

**Published:** 2019-02-20

**Authors:** Tyler J. Maiers, Daniel C. Wang, Ali H. Houjaij, Oussama M. Darwish

**Affiliations:** ^1^Western New York Healthcare System-Buffalo Veterans Affairs Medical Center, 3495 Bailey Ave, Buffalo, NY 14215, USA; ^2^Department of Urology, Jacobs School of Medicine and Biomedical Sciences, University at Buffalo, Buffalo General Medical Center, Suite B280, 100 High Street, Buffalo, NY 14203, USA

## Abstract

Renal oncocytomas and retroperitoneal schwannomas are rare and typically benign tumors with characteristic histopathologic features. Ideal management of both renal oncocytoma and retroperitoneal schwannoma is surgical resection. We present a rare case of a 63-year-old man with multifocal renal oncocytoma and retroperitoneal ancient schwannoma which, preoperatively, masqueraded as metastatic renal cell carcinoma. Both tumors were successfully resected surgically. Immunochemistry and histopathology confirmed each diagnosis.

## 1. Introduction

Renal oncocytoma is a rare tumor accounting for approximately 4% of all solid renal masses [1]. While the majority of oncocytomas are benign, there have been several case reports of malignant behavior and metastasis [2, 3]. Oncocytomas are composed of oncocytes, which are uniform, round, or polygonal neoplastic cells exhibiting a granular eosinophilic cytoplasm. Long-term outcomes in patients with oncocytomas are highly favorable, with previous reports indicating a disease-specific survival of up to 100% [4].

Similarly, primary tumors of the retroperitoneum are uncommon. Schwannomas are thought to occur in approximately 4% of all primary retroperitoneal tumors [5]. Schwannomas originate in peripheral nerve fibers and are typically found in the head, neck, or flexor surfaces of the extremities. Although malignant schwannomas have been reported in the literature [6], the vast majority are benign in nature. The term “ancient” schwannoma is reserved for schwannomas exhibiting signs of degeneration including hemorrhage, cyst formation, hyalinization, and calcification [7].

To the best of our knowledge, the present study reports the first case of multifocal renal oncocytoma and retroperitoneal ancient schwannoma. The patient was treated with open right radical nephrectomy and retroperitoneal lymph node dissection. The clinical, radiographic, and pathologic findings of this case are discussed in detail.

## 2. Case Presentation

**Figure 1:**
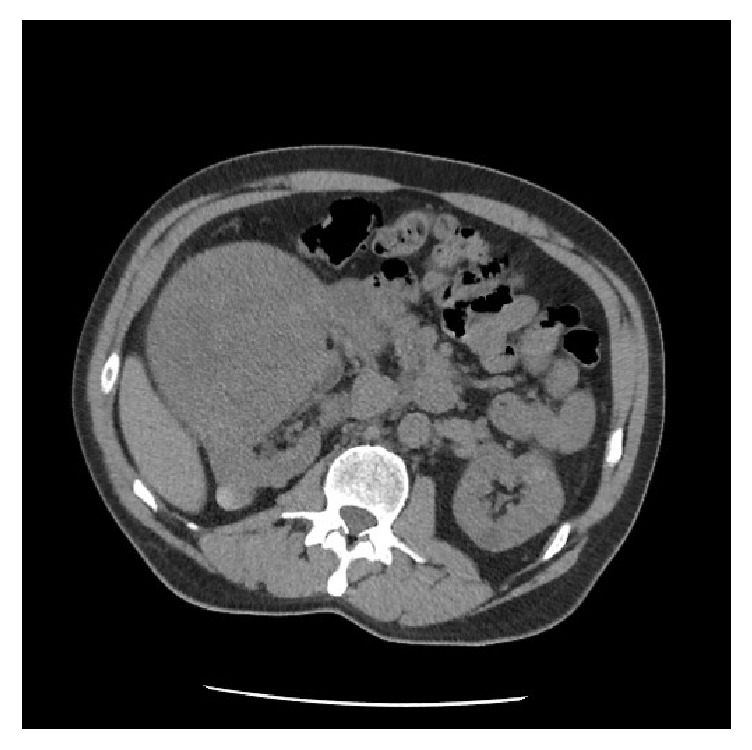
Computed tomography without contrast revealed a large right renal mass with significant mass effect on the right kidney. Adjacent soft tissue nodularities were concerning for satellite nodules or metastatic disease.

**Figure 2:**
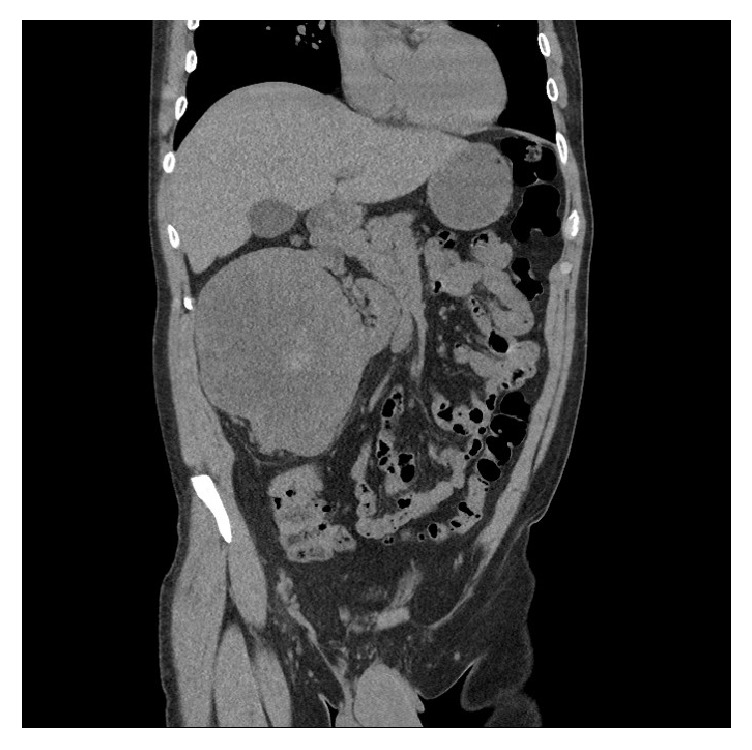
Computed tomography without contrast demonstrating a large heterogeneous right renal mass.

**Figure 3:**
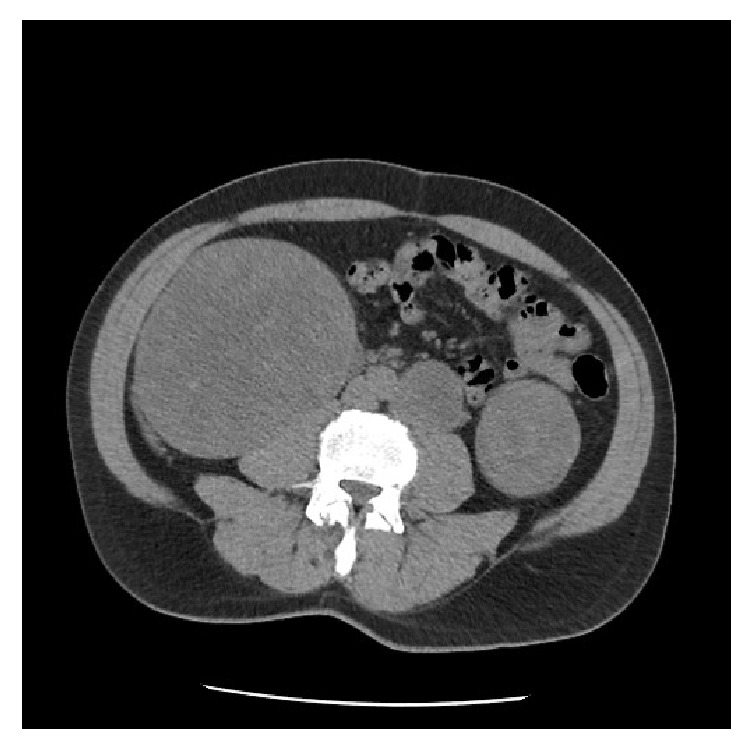
Computed tomography without contrast demonstrating left paraaortic mass, left interpolar renal mass, and large right renal mass.

A 63-year-old man with chronic kidney disease presented with elevated baseline creatinine. He had no urologic symptoms and no history of flank pain or hematuria. Family history was notable for renal malignancy in the patient's grandmother. The physical examination was unremarkable, with no palpable flank mass or tenderness. Laboratory studies were notable for a creatinine of 2.02 (eGFR = 34 ml/min), up from a baseline of 1.60 (eGFR = 42 ml/min). Renal ultrasound revealed a 12 x 15 cm predominantly solid mass in the right kidney with internal cystic changes and central flow. In the left kidney renal ultrasound revealed a solid-appearing mass in the upper pole measuring 6.5 x 6 x 5.5 cm, a hypoechoic structure measuring 4.8 x 4.1 x 4.6 cm in the lower pole, and an adjacent 6.1 x 5.8 x 6.4 cm solid left lower pole renal mass with a small amount of central flow. Computed Tomography (CT) revealed a 14 x 13 x 16 cm right renal mass almost completely replacing the interpolar region, with significant mass effect on the right kidney (Figures 1 and 2). Adjacent tissue nodularity in the perinephric fat was concerning for satellite nodules or metastatic disease (Figure 1). Right retroperitoneal adenopathy measuring 2.4 x 2.7 cm at the level of the renal hilum was identified. In addition, multiple 2-3 cm hyper- and isodense indeterminate soft tissue lesions were identified in the right kidney. The left kidney was notable for multiple solid renal masses measuring 6.3 cm at the upper pole and 4.6 cm at the interpolar region (Figure 3). A left paraaortic soft tissue mass measuring 4.6 x 4.6 cm with associated calcification was concerning for adenopathy (Figure 3). Whole body positron emission tomography (PET)/CT imaging was obtained to evaluate for metastatic disease. PET/CT revealed bilateral metabolically active solid renal masses concerning for malignancy and metabolic activity in a left paraaortic soft tissue mass concerning for lymphadenopathy. Magnetic resonance imaging (MRI) revealed no evidence of inferior vena cava thrombus.

The patient was discussed in a multidisciplinary oncology meeting and the decision was made to proceed with surgical extirpation of the right kidney and the paraaortic mass. We performed an open right radical nephrectomy with right retroperitoneal lymph node dissection and excision of left paraaortic mass.

**Figure 4:**
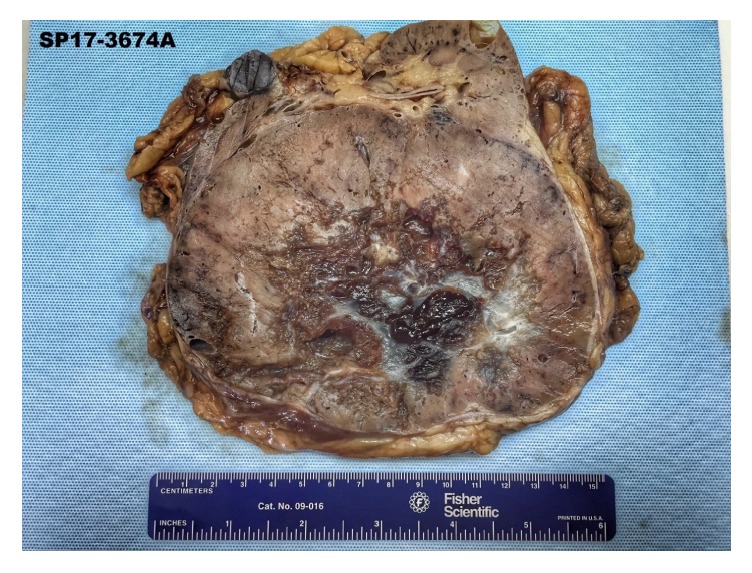
Gross specimen of right kidney containing maroon-colored mass with gray-white central scar.

**Figure 5:**
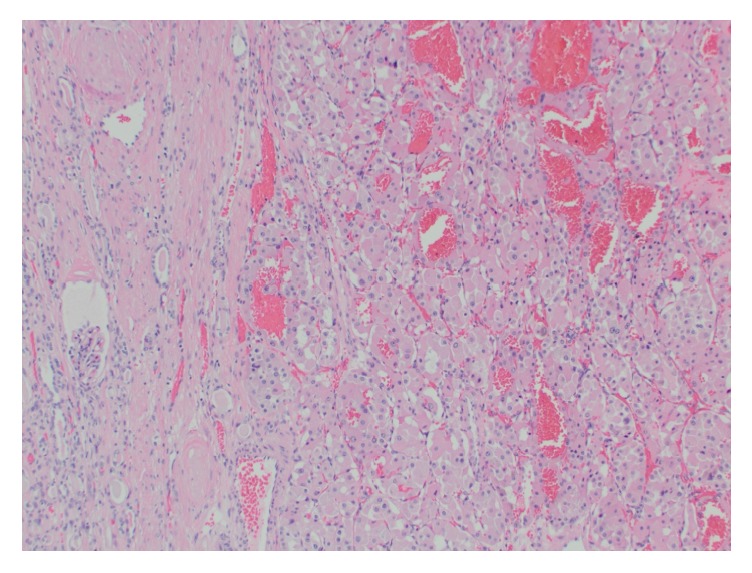
Microscopic (100x) H & E stained tumor revealing nests of round and polygonal cells with granular, eosinophilic cytoplasm classic for oncocytoma.

**Figure 6:**
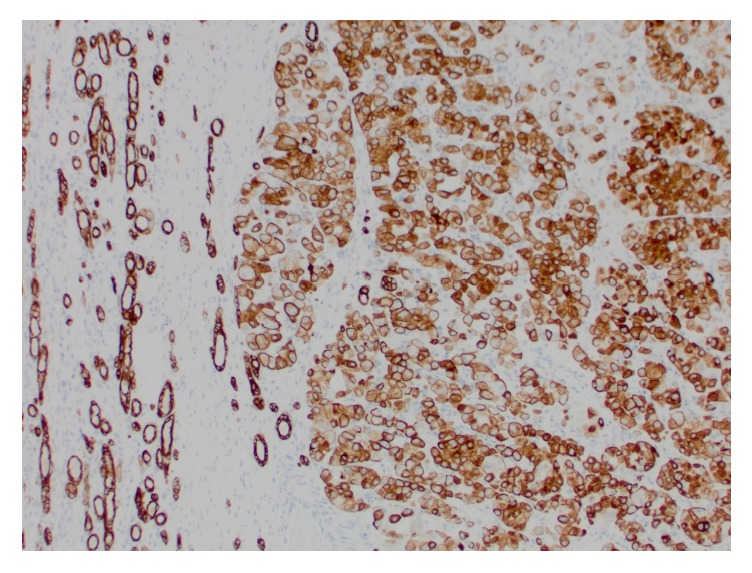
Microscopic (40x) renal tumor cells demonstrated positive staining for CK7.

Grossly, the right kidney contained a spherical, encapsulated, and homogenous maroon-colored mass with a gray-white lobulated central scar (Figure 4). Two additional smaller masses with similar gross appearance but without central scar were also present. Histologic examination revealed nests of round and polygonal cells with granular, eosinophilic cytoplasm (Figure 5) consistent with oncocytoma. Immunohistochemical staining of the right renal mass with colloidal iron, CD117, CK7, RCC, and CD10 was performed. The tumor cells were positive for CD117, CK7 (Figure 6), and weakly positive for CD10. The tumor cells were negative for Hale colloidal iron and RCC.

**Figure 7:**
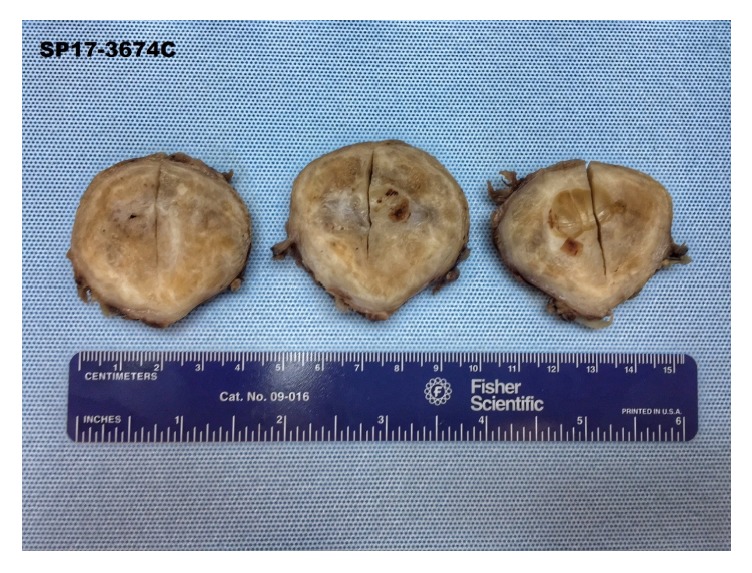
Cross sections of retroperitoneal ancient schwannoma with glistening tan-white surface.

Cross sections of the left paraaortic mass revealed a smooth, encapsulated mass with a pearly and glistening tan-white surface (Figure 7). Histologic examination revealed multiple regions of cystic degeneration, positive staining for S100, and Antoni A and Antoni B areas. The final pathologic diagnoses were renal oncocytoma and left paraaortic ancient, benign schwannoma.

## 3. Discussion

Renal oncocytoma is a benign tumor that is often thought to represent renal cell carcinoma during the preoperative workup due to similar enhancement on imaging. Distinguishing between the two is critically important yet consistently challenging for the practicing urologist. The similar rate of growth, age at presentation, and peak incidence during the seventh decade further complicate the task of distinguishing the two from one another. This case demonstrates the inherent difficulty in distinguishing preoperatively the difference between metastatic renal cell carcinoma and a combination of rare, typically benign tumors of the kidney and retroperitoneum.

The gross appearance of an oncocytoma is described as a homogeneous tan or mahogany mass with a pseudocapsule and central scar. Microscopically, oncocytomas are characterized by nests of round and polygonal, highly eosinophilic cells with granular cytoplasm secondary to a high concentration of mitochondria [2]. Oncocytomas are found more commonly in men than women (1.2:1), and multifocal oncocytomas are identified in up to 13% of patients with oncocytoma [2]. Cytogenetic abnormalities used to identify an oncocytoma include a diploid karyotype, loss of chromosomes 1, loss of Y, or various rearrangements of 11q13 [8]. Immunohistochemical characteristics used to identify oncocytoma include positive staining for CK7, CD117, negative staining for vimentin, and negative or luminal staining for Hale colloidal iron [8]. The eosinophilic variant of chromophobe renal cell carcinoma represents perhaps the most histologically similar variant to oncocytoma. Specific cutoffs for distinguishing between oncocytoma and the eosinophilic variant of chromophobe renal cell carcinoma on the basis of CK7 positivity are highly variable and not well agreed upon in the literature [8]. Despite the inherent difficulty in confirming a diagnosis, distinguishing between these two entities is critical as the incidence of coexisting renal cell carcinoma and oncocytoma in patients with multiple renal tumors is approximately 10% [9]. When multiple renal tumors are present as in the patient described in this case, the urologist must maintain a high index of suspicion for coexisting renal cell carcinoma.

A number of subtle imaging findings have been described in an attempt to differentiate oncocytoma from renal cell carcinoma on CT and MRI. On CT, oncocytomas typically have a high peak Hounsfield unit attenuation in the nephrogenic phase as opposed to the corticomedullary phase most often seen in renal cell carcinomas. A form of segmental enhancement inversion on CT has been proposed as a unique method to distinguish between small oncocytomas and renal cell carcinoma [10]. A later study comparing segmental enhancement inversion on MRI, among a variety of other features, found no reliable characteristics to use in distinguishing between renal oncocytoma and chromophobe renal cell carcinoma [11]. Unfortunately for the practicing urologist, distinguishing the two entities radiographically remains challenging.

In this case, the identification of a solid retroperitoneal mass was concerning preoperatively for metastatic disease. The retroperitoneum can host a wide range of benign and malignant neoplasms. Schwannomas are composed of Schwann cells arising from a peripheral nerve sheath. Characteristic findings include S100 positivity and distinct regions of hypercellularity and hypocellularity, termed Antoni A and Antoni B regions, respectively. Ancient schwannoma is an uncommon variant used to describe a schwannoma with degenerative changes mimicking a malignant tumor including hemorrhage, cystic necrosis, calcification, and hyalinization [7]. The specimen obtained in this case contained multiple regions of cystic degeneration consistent with the ancient schwannoma variant. Complete surgical excision of a schwannoma is recommended and portends a good prognosis [7]. Approximately 11.7% of all schwannomas excised with a wide surgical margin are found to recur, with higher rates of recurrence reported following partial excision or enucleation [12].

Renal mass biopsy was discussed extensively at a multidisciplinary oncology conference before deciding upon the ultimate treatment strategy in this case. The large right renal tumor exhibited significant mass effect on the right kidney and replaced nearly the entire interpolar region with localized extension into perinephric fat. Definitive surgical management was thus considered optimal irrespective of biopsy results. Furthermore, the right renal mass was considered the most likely candidate to represent primary tumor in the setting of possible metastatic disease. The left renal masses, by comparison, were significantly smaller in size with fewer concerning features. Preoperatively, there was concern for a single metastatic site to the retroperitoneum. In the setting of perceived oligometastatic disease in a patient with ECOG performance status of 1, we elected to pursue a cytoreductive nephrectomy via surgical extirpation of the right kidney and excision of the left paraaortic mass.

Despite an extensive preoperative workup highly concerning for metastatic renal cell carcinoma, this patient was found to have separate, benign lesions with a favorable overall prognosis. To the best of our knowledge, this is the first described case of combined multifocal renal oncocytoma and retroperitoneal ancient schwannoma masquerading as metastatic renal cell carcinoma. Due to the possibility of a hybrid tumor on the left consisting of chromophobe renal cell carcinoma and oncocytoma, planned follow-up includes CT imaging every six months to assess for interval growth. Follow-up CT imaging at six months revealed no interval growth and no evidence of metastasis.
